# Connectivity Gradient in the Human Left Inferior Frontal Gyrus: Intraoperative Cortico-Cortical Evoked Potential Study

**DOI:** 10.1093/cercor/bhaa065

**Published:** 2020-03-31

**Authors:** Takuro Nakae, Riki Matsumoto, Takeharu Kunieda, Yoshiki Arakawa, Katsuya Kobayashi, Akihiro Shimotake, Yukihiro Yamao, Takayuki Kikuchi, Toshihiko Aso, Masao Matsuhashi, Kazumichi Yoshida, Akio Ikeda, Ryosuke Takahashi, Matthew A Lambon Ralph, Susumu Miyamoto

**Affiliations:** 1 Department of Neurosurgery, Kyoto University Graduate School of Medicine, Kyoto 606-8507, Japan; 2 Department of Neurology, Kyoto University Graduate School of Medicine, Kyoto 606-8507, Japan; 3 Epilepsy Center, Cleveland Clinic, Cleveland, OH 44106, USA; 4 Laboratory for Brain Connectomics Imaging, RIKEN Center for Biosystems Dynamics Research, Hyogo 650-0047, Japan; 5 Department of Epilepsy, Movement Disorders and Physiology, Kyoto University Graduate School of Medicine, Kyoto 606-8507, Japan; 6 MRC Cognition and Brain Sciences Unit, University of Cambridge, Cambridge CB2 7EF, UK; 7 Department of Neurosurgery, Shiga General Hospital, Moriyama, Shiga 524-0022, Japan; 8 Division of Neurology, Kobe University Graduate School of Medicine, Kobe, Hyogo 650-0017, Japan; 9 Department of Neurosurgery, Ehime University Graduate School of Medicine, To-on, Ehime 791-0295, Japan

**Keywords:** 4D visualization, cortico-cortical evoked potential, dual-stream language model, fronto-temporal radiation

## Abstract

In the dual-stream model of language processing, the exact connectivity of the ventral stream to the anterior temporal lobe remains elusive. To investigate the connectivity between the inferior frontal gyrus (IFG) and the lateral part of the temporal and parietal lobes, we integrated spatiotemporal profiles of cortico-cortical evoked potentials (CCEPs) recorded intraoperatively in 14 patients who had undergone surgical resection for a brain tumor or epileptic focus. Four-dimensional visualization of the combined CCEP data showed that the pars opercularis (Broca’s area) is connected to the posterior temporal cortices and the supramarginal gyrus, whereas the pars orbitalis is connected to the anterior lateral temporal cortices and angular gyrus. Quantitative topographical analysis of CCEP connectivity confirmed an anterior–posterior gradient of connectivity from IFG stimulus sites to the temporal response sites. Reciprocality analysis indicated that the anterior part of the IFG is bidirectionally connected to the temporal or parietal area. This study shows that each IFG subdivision has different connectivity to the temporal lobe with an anterior–posterior gradient and supports the classical connectivity concept of Dejerine; that is, the frontal lobe is connected to the temporal lobe through the arcuate fasciculus and also a double fan-shaped structure anchored at the limen insulae.

## Introduction

Language is a unique feature of human beings that should not be impaired by surgery without the justification of clinical benefit. The posterior part of the inferior frontal gyrus (IFG), which consists of the pars opercularis (pOpe) and pars triangularis (pTri), is known as Broca’s area, while the anterior part, the pars orbitalis (pOrb), is reported to have an executive function in semantic cognition tasks (Wagner et al. 2001; Gough et al. 2005; Hoffman et al. 2010; Krieger-Redwood et al. 2015). Recently, a dual-stream model of language processing has been proposed and received wide recognition on the strength of an analogy with the processing of visual information (Hickok and Poeppel 2004, 2007; Saur et al. 2008; Ueno et al. 2011; Hickok 2012; Gil-Robles et al. 2013). The model consists of a dorsal stream for phonological processing and a ventral stream for semantic processing. Although this framework is generally accepted, the details of the tracts and cortices involved in the dual network remain to be established (Dick and Tremblay 2012). While the superior longitudinal fasciculus (SLF) and arcuate fasciculus (AF) are established as the main pathway of the dorsal network, additional studies are required to identify the connectivity underlying the ventral network, which presumably includes the uncinate fasciculus (UF), the inferior longitudinal fasciculus (ILF), and the inferior fronto-occipital fasciculus (IFOF).

Among these, the IFOF is reported to be involved in semantic processing based on high-frequency electrical stimulation of the white matter in awake surgery (Duffau et al. 2005). The IFOF consists of two components, superficial and deep. The former originates from the anterior part of the IFG (pOrb and pTri), and the latter originates from the dorsolateral prefrontal cortex, middle frontal gyrus (MFG), and orbitofrontal cortex (Sarubbo et al. 2013). Their posterior termination includes the occipital lobe, superior parietal lobule (SPL), and posterior part of the temporo-basal area (Martino et al. 2010a). Duffau and coworkers reported that stimulation of the superficial component of the IFOF induced semantic errors during picture naming. The supposition that the IFOF engages in semantic processing is also supported by functional studies on its cortical terminations. The anterior part of the IFG, which is part of the frontal termination of the superficial component of the IFOF, has been shown to be engaged in semantic control by a meta-analysis of functional magnetic resonance imaging (fMRI) and positron-emission tomography (PET) studies (Noonan et al. 2013) and by interventions using transcranial magnetic stimulation (Hoffman et al. 2010; Jefferies 2013). The posterior fusiform gyrus, which is one of the posterior terminations of the IFOF, is known as the visual word form area. A role for the IFOF in semantic processing is further supported by dynamic causality modeling of blood oxygenation level dependent signals that showed effective connectivity from the fusiform gyrus to the anterior IFG during a semantic judgment task (Perrone-Bertolotti et al. 2017). These lines of evidence suggest that the superficial component of the IFOF has a semantic function. However, the anterior temporal lobe (ATL), which engages in semantic representation and is essential in semantic cognition (Spitsyna et al. 2006; Lambon Ralph et al. 2010; Jefferies 2013; Lambon Ralph et al. 2017), has not been reported as a posterior termination of the IFOF. ATL subregions receive the terminations of the UF (temporal pole) and ILF (anterior-ventral area and temporal pole) (Binney et al. 2012; Fan et al. 2014; Egger et al. 2015; Jung et al. 2017; Panesar et al. 2018) but seem not to be a main part of the ventral spoken language stream. Electrical stimulation of the UF does not interfere with object naming, and resection of the UF is generally acceptable in neurosurgery (Duffau et al. 2008, 2009). The ILF projects posteriorly to the occipital lobe without any frontal termination. If the superficial component of the IFOF and the anterior part of the IFG are implicated in semantic function, it would be natural to infer that the ATL, which is the semantic representational hub, has a direct connection to the semantic control center (the anterior part of the IFG) via a subcomponent of the IFOF, although no termination of the IFOF in the ATL has been proven. To verify this hypothesis, we investigated in detail the connectivity between the IFG and the lateral temporal cortices.

**Table 1:** Demographic features of the patients

Patient no., age, sex, handedness	Language-dominant side	Surgery side	Etiology	Pathology	Recording electrodes, n	Stimulus sites, n	Stimulus intensity (mA)	Coverage of stimulus sites (frontal)	Coverage of stimulus sites (temporal)	Coverage of stimulus sites (parietal)
1, 40, F, R	L	L	Tumor	Diffuse astrocytoma	30	20	15	IFG, PreCG	STG, MTG, ITG	SMG, PostCG
2, 74, F, R	—^a^	L	Tumor	Glioblastoma	30	12	15	IFG, PreCG	STG, MTG, ITG	—
3, 35, F, R	L	L	Tumor	Glioblastoma	32	15	15	IFG, PreCG, MFG	MTG, ITG	—
4, 79, M, R	L^b^	L	Tumor	Glioblastoma	32	17	15	IFG, PreCG	STG, MTG, ITG	SMG, AG
5, 64, M, R	L^b^	L	Tumor	Glioblastoma	64	15	15	IFG, PreCG	STG, MTG, ITG	SMG, AG, TPJ
6, 21, M, R	L	L	Epilepsy	FCD type Ia	30	22	15	IFG, PreCG	STG, MTG, ITG	—
7, 32, F, R	L	L	Tumor	Anaplastic astrocytoma	32	8	15	IFG, PreCG	STG, MTG	—
8, 25, M, R	L	L	Tumor	Dysembryoplastic neuroepithelial tumor	31	11	15	IFG, MFG	STG, MTG	SMG
9, 31, F, R	L	L	Tumor	Diffuse astrocytoma	32	15	15	IFG, PreCG, OFC	STG, MTG	SMG, AG, TPJ
10, 43, M, R	L	L	Tumor	Anaplastic oligoastrocytoma	27	11	15	IFG, PreCG, MFG, FP	STG, MTG	—
11, 62, M, R	L	L	Tumor	Anaplastic oligoastrocytoma	30	8	15	IFG, PreCG, MFG	MTG	—
12, 45, F, R	L	L	Tumor	Anaplastic astrocytoma	50	22	15	IFG, PreCG	STG, MTG, ITG	AG
13, 50, F, R	L	L	Epilepsy	Hippocampal sclerosis	32	5	15	IFG, PreCG	—	—
14, 42, M, R	L	L	Tumor	Glioblastoma	44	7	15	IFG, PreCG	—	AG

Note: In the column “language-dominant side,” the “a” indicates the case in which the WADA test and language fMRI was omitted because the patient had definite motor aphasia caused by the lesion. The “b” indicates the cases in which the WADA test was omitted because the clinical situation was urgent. In both cases, fMRI showed left dominance of language. PreCG, precentral gyrus; PostCG, postcentral gyrus; STG, superior temporal gyrus; ITG, inferior temporal gyrus; and TPJ, temporoparietal junction.

The cortico-cortical evoked potential (CCEP) is an electrophysiological tool that is used to probe effective connectivity by applying single-pulse electrical stimulation to the cortex. CCEPs are recorded from remote cortical areas and are presumed to reflect orthodromic propagation of the stimulus signal through the cortico-cortical connections (Matsumoto et al. 2017; Matsumoto and Kunieda 2018). This method was first applied in patients with implanted subdural electrodes and successfully delineated various functional cortical networks (Matsumoto et al. 2004; Lacruz et al. 2007; Conner et al. 2011; Koubeissi et al. 2012; Swann et al. 2012; Kubota et al. 2013; Matsuzaki et al. 2013; Keller et al. 2014a; Enatsu et al. 2015; Usami et al. 2018). The connectivity pattern retrieved as CCEPs overlaps in large part with the resting-state functional connectivity measured by resting-state fMRI (Keller et al. 2011, 2014a). Due to its high practicality and reproducibility, CCEP has been clinically utilized to probe and monitor the connectivity of the AF during neurosurgical operations (Saito et al. 2014; Yamao et al. 2014; Yamao et al. 2017) to ensure speech preservation (known as “intraoperative CCEP” examination).

To investigate the patterns of connectivity from the IFG to the temporoparietal area, we systematically applied single-pulse stimulation to three IFG subdivisions (pOrb, pTri, and pOpe) and recorded the CCEP responses in an intraoperative setting. Although the connectivity between the posterior IFG and the inferior parietal and posterior temporal areas has been intensively studied (Greenlee et al. 2004; Matsumoto et al*.* 2004; Greenlee et al. 2007; Garell et al. 2013; Yamao et al. 2014), this study is unique in that we visualized and analyzed the spatiotemporal dynamics of CCEP connectivity from all the IFG subdivisions, in particular from the anterior IFG, in light of the dual-stream model of language processing.

## Materials and Methods

### Participants

**Figure 1:**
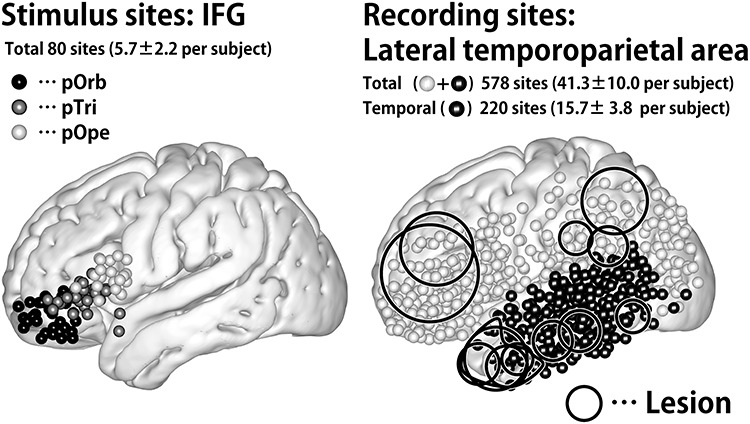
Distribution of stimulus and recording sites. (Left) Shown are the stimulus sites in all patients plotted in MNI space. The coordinate of a stimulus site was defined as the midpoint of the stimulation electrode pair. The black, gray, and white spheres indicate the location of the stimulus sites in IFG pOrb, pTri, and pOpe, respectively. (Right) The recording sites in all patients plotted as spheres in MNI space. The black spheres indicate sites in the temporal lobe, and the white ones indicate those out of the temporal lobe in individual brains. The circles indicate the location of the lesions, removal of which was the indication for surgery. The lesions are widely distributed in the frontal, parietal, and temporal opercula except for the insular cortex because we excluded those patients who had a lesion or intense edema in the temporal stem.

Fourteen patients (7 male; mean age, 45.9 ± 17.2 years) were recruited for this study. The patient demographics are shown in Table 1. These patients were selected from 49 consecutive patients who underwent surgical resection of a cerebral lesion in the language-dominant hemisphere between March 2014 and July 2016. All the selected patients were supplied with the appropriate information about the study, provided written informed consent, and received intraoperative CCEP. The inclusion criteria were as follows: (1) the stimulus sites of the CCEP investigation covered all three subdivisions of the IFG (pOrb, pTri, and pOpe), which was confirmed by intraoperative photographs of the grid electrodes; (2) the recording electrodes covered the lateral temporo-parietal area; and (3) no invasion or severe mass effect was observed in the temporal stem or extreme capsule where the fibers of the ventral stream converge (see Fig. 1).

In 11 patients, the language-dominant hemisphere was determined by the WADA test with intracarotid administration of propofol (Takayama et al. 2004). In two cases (patients 4 and 5), the WADA test was omitted because of the urgent clinical situation and language fMRI was used instead to define the dominant hemisphere (Yamao et al. 2014). In one case (patient 2), no language tasks could be performed because of motor aphasia resulting from the tumor, indicating that the affected hemisphere was language-dominant. Intraoperative CCEP investigation was performed to monitor the integrity of the dorsal language pathway during surgery. The clinical usefulness of monitoring the dorsal language pathway has been reported in the literature (Yamao et al. 2014).

This study was approved by the Ethics Committee of Kyoto University Graduate School and the Faculty of Medicine (IRB C573, C443, and C1082).

### Preparation and Acquisition of Intraoperative CCEP Data

The craniotomy was performed under general anesthesia in all patients. After the dura was opened, one grid electrode was placed on the frontal lobe to cover the anterior language core (Broca’s area) and another one or two grid electrodes were placed on the tempo-parietal cortices to cover the posterior language core (Wernicke’s area). The location of electrodes was always determined by clinical need (i.e., monitoring of CCEP and functional mapping).

For the preoperative planning of grid placement, anatomical and functional images were acquired with a 3-Tesla magnetic-resonance scanner (Trio, Siemens, Erlangen, Germany) as described elsewhere (Yamao et al. 2014, 2017). We constructed a cortical surface model from the T1-weighted image using FreeSurfer software (https://surfer.nmr.mgh.harvard.edu/).

The electrodes were made of platinum with a recording diameter of 3 mm and a center-to-center distance of 1 cm (Unique Medical Co., Ltd, Tokyo, Japan). The details of CCEP recording have been reported elsewhere (Matsumoto et al. 2004, 2007, 2012; Yamao et al. 2014). A 32-channel intraoperative monitoring system (MEE 1232 Neuromaster, Nihon-Kohden, Tokyo, Japan) equipped with an electrical stimulator (MS-120B, Nihon-Kohden, Tokyo, Japan) was used to generate single pulses for stimulation and record the raw electrocorticogram (ECoG) and for online analysis of the averaged CCEP waveform. The raw ECoG data were recorded at a sampling rate of 5 kHz. Recordings from subdural electrodes were referenced to a scalp electrode placed on the skin over the mastoid process contralateral to the side of craniotomy.

Single-pulse electrical stimulation was applied in a bipolar fashion using a pair of adjacent electrodes. Square-wave electrical pulses of alternating polarity with a pulse width of 0.3 ms were delivered at 1 Hz. We fixed the stimulation intensity at 15 mA to shorten the investigation time given that we did not have enough time to adjust the stimulus intensity in every session in the operating room. The stimulation order was as follows. First, we stimulated all possible electrode pairs in the IFG while recording the CCEP responses in the temporo-parietal area. We then stimulated selected temporo-parietal electrodes, namely those with discrete CCEP responses to IFG stimulation, to investigate the reciprocal connectivity. All the CCEP responses analyzed in this study were recorded under general anesthesia. CCEP examination under general anesthesia was feasible because the distribution of the CCEP response does not change even though the amplitude of the maximum response becomes slightly larger in the awake state (Yamao et al. 2014).

The online CCEP analysis was obtained by averaging the ECoGs (30 trials in each session) across time windows phase-locked to stimulation, each with a poststimulus duration of 200 ms and a prestimulus baseline of 20 ms. We checked the reproducibility of the response in at least two sessions to distinguish the CCEP responses from the baseline values. The raw ECoG was simultaneously recorded and displayed to monitor seizure patterns during stimulation. The online CCEP analysis was used to determine the stimulation electrodes to be used for testing reciprocal connectivity. The recorded raw ECoG data were used for further offline analysis. The offline analysis was obtained by averaging ECoGs phase-locked to the stimuli (30 trials per session) with a time window of 300 ms and a baseline of 30 ms before stimulus onset.

### Visualization of Spatiotemporal Dynamics of the CCEP: The 4D CCEP Map

Obtaining a clear understanding of the whole connectivity pattern just from an inspection of the waveforms of individual patients is difficult because the electrode locations differ from patient to patient. To understand the spatiotemporal dynamics based on the data derived from all patients, we created a “4D CCEP map,” that is, a 4D (time-sequence of 3D) volume image in the Montreal Neurological Institute (MNI) standard space, creating one such map for each stimulus area in the IFG (pOrb, pTri, and pOpe). For each time point (time-locked at a stimulation), all amplitude data were plotted in the MNI space, smoothed with a Gaussian kernel to visualize point data in 3D space, and averaged across all sessions (the technical details are described in the Supplementary Materials). Uneven electrode coverage was corrected by incorporating the electrode density information across the patients. To visualize the 4D representation of the CCEP, we digitally rendered a standard brain surface model, providing each vertex with the value at the nearest-neighbor voxel in the 4D CCEP map. The time sequence of this rendered brain surface is presented as a movie (available in the Supplementary Materials).

### Topographical Analysis of Frontotemporal Connectivity

To clarify the spatial relationships between the stimulus and response sites, we performed a linear regression analysis on their coordinates, i.e., a “topographical analysis” of the CCEP. First, we determined the CCEP response by visual inspection of the waveforms using the following criteria:

1. The polarity is negative.
2. The amplitude is larger than 6× the standard deviation (SD) of the baseline fluctuations; the baseline is defined as the period between 100 and 5 ms prestimulus.
3. The response is reproducible across two consecutive sessions (30 trials are averaged for each session).

We excluded data from electrodes located within 25 mm of the stimulus site to rule out responses due to local U-fibers because our objective was to investigate the long-range CCEP responses. Volume-conducted responses, although rare in areas >25 mm distant from the stimulus site, were eliminated by visual inspection because they putatively reflect large responses just under the stimulus area (Shimada et al. 2017). We judged responses to be volume-conducted when the waveforms were almost invariant in shape and diminished steadily with distance from the stimulus site. After we inspected all the recorded waveforms to determine the early and delayed CCEP responses, the basic properties of the CCEP responses, such as onset time, peak time, and amplitude, were stored in a database (referred to hereafter as the CCEP database) together with the MNI coordinates of the electrodes. We classified each response as early (N1) or delayed (N2) by a cluster analysis of the latency distribution (see Supplementary Figure 1), although the N1 cluster determined in this method was similar to the traditional criteria of N1 (onset < 30 ms, peak < 100 ms). We focused on N1 rather than N2 because N1 is supposed to reflect orthodromic propagation via cortico-cortical connections (Matsumoto et al*.* 2017; Matsumoto and Kunieda 2018).

Although we judged the CCEP response based solely on single waveforms, traditional waveform analysis has paid attention to locally maximal responses that seem to be the center of the response when adjacent electrodes show a similar waveform. To perform a similar analysis in this study for purposes of comparison, we identified maximum response sites in the CCEP database automatically using a MATLAB script written in-house. We defined a “max response” site as one that had the largest amplitude in the spatio-temporal neighborhood, where spatial proximity means within 15 mm of the interelectrode distance and temporal proximity means within 5 ms of the peak time difference.

After collating the CCEP database, we investigated whether the spatial distribution of N1 responses in the temporo-parietal area differs according to the stimulus site in the IFG. Given that the distribution of the response sites is parallel to the *y*–*z* plane, we verified the difference in the 2D distribution (of MNI *y* and *z* coordinates) using Wilk’s lambda test. We also evaluated the hypothesis that the more anterior the location of the stimulus site (in IFG), the more anterior the response site (in the lateral temporal cortices). We created new coordinates for the stimulus and response sites separately. We measured the distance between the stimulus site and the midpoint of the lower third of the precentral sulcus (see Fig. 4*c*, left panel) for the stimulus sites. We performed principal component analysis (PCA) for all the N1 response sites and extracted the anterior–posterior axis parallel to the temporal gyri as the first component (named Y1; see Fig. 4*c*) for the response sites. The second component indicates the direction perpendicular, i.e., in a dorso-ventral temporal lobe orientation, to Y1 (named Y2). We performed linear regression analysis of X and Y1 or Y2.

### Analysis of Fronto-Parietal Connectivity

Given that the number of cases that covered the parietal area was small and the statistical analysis described above was not feasible, we only described the area-to-area connectivity by detecting the maximum response electrodes in each CCEP examination. We planned to perform a similar topographical analysis between the IFG and parietal lobe. Here, we focused on pOrb connectivity to the inferior parietal area as investigated by pOrb stimulation because previous literature has reproducibly reported the CCEP connectivity between pOpe/pTri and the inferior parietal area (Matsumoto et al. 2012; Entz et al. 2014; Keller et al*.* 2014b; Yamao et al*.* 2017).

### Probing Reciprocal Connections From and to the Pars Orbitalis

We investigated the reciprocality of the connections from pOrb rather than pTri or pOpe because the latter has already been investigated in our previous reports using a different patient population (Matsumoto et al. 2004; Yamao et al*.* 2017). We judged a connectivity to be reciprocal when CCEP revealed a bidirectional relationship between the stimulus and response sites. Given that we stimulated IFG in a comprehensive manner, we first extracted frontotemporal connectivity (which indicates a relationship between a frontal stimulus site and a temporal response site; abbreviated as “F→T connectivity” hereafter) from the CCEP database and then investigated whether the stimulus and response sites still have CCEP connectivity if we interchange the stimulus site and response site. We calculated the reciprocality rate of the fronto-temporal (F→T) and fronto-parietal (F →P) connectivity separately. The reciprocality rate was defined as the proportion of the reciprocal connections in the sessions stimulating through the F→T (or F→P) response sites. The technical details regarding this procedure are provided as Supplementary Materials. We calculated the reciprocality rate for six groups of stimulus-recording pairs stratified by area (F→T vs. F→P) and type of response (max response, any response, or no response). We treated the stimulus-recording pair with no CCEP response in the same way as those with a CCEP response in order to obtain the negative controls.

### Comparison with Resting-State fMRI Connectivity

We compared the CCEP connectivity originating from the IFG pOrb with the functional connectivity revealed by the resting state (rs)-fMRI for the purpose of validation. We utilized the functional connectivity maps available from the NeuroSynth website (http://neurosyngh.org/) derived from 1000 healthy subjects as a reference. For each stimulus site in IFG pOrb, a functional connectivity map was obtained from the website as a 3D volume image by specifying the stimulus coordinate as the seed voxel. We calculated the voxel-wise average of all functional connectivity values obtained, as described above for one volume image. We subsequently visualized the averaged connectivity map as a color map on the standard brain surface and compared it with the CCEP map by visual inspection.

**Figure 2:**
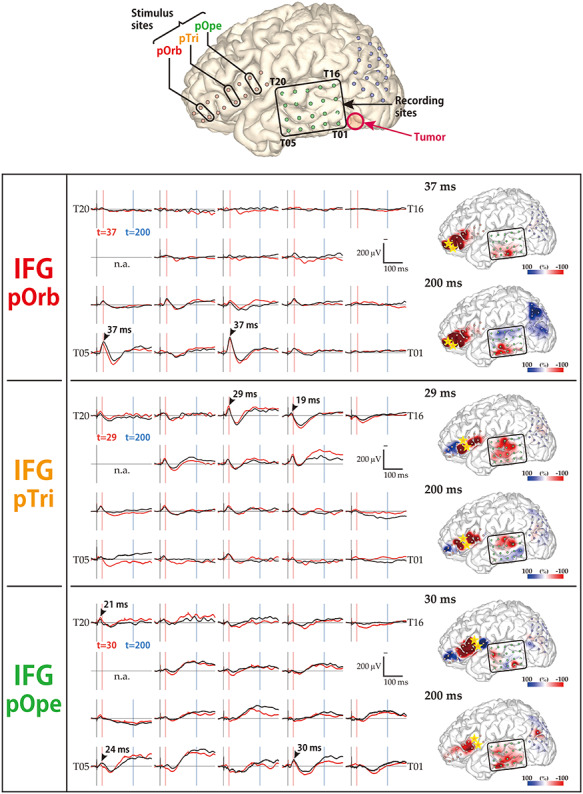
Diversity in the response pattern across stimulus sites (patient 12). (Row 1) Shown are the locations of all electrodes in patient 12. A 2 × 8 electrode grid was placed on the IFG (contains Broca’s area), and a 4 × 5 electrode grid was placed on the temporal lobe and on the parietal lobe (jointly containing Wernicke’s area). The open red circle indicates the location of the tumor. (Rows 2–4) Shown are the response patterns due to stimulation in pOrb, pTri, and pOpe, respectively. Each row corresponds to one stimulus site in the IFG. (Left) Shown are 19 of the 20 waveforms recorded by the temporal grid, with upward being more negative. The lines in black and red represent the averaged waveforms of two consecutive sessions of 30 trials each. The black arrowheads indicate the “maximum response” sites and the peak latencies at those sites. The red and blue vertical lines indicate the timing of typical early and delayed responses, respectively. (Right) Shown are a pair of brain surface models painted by amplitude in the early and delayed phases, respectively. Negative amplitudes are red and positive amplitudes are blue. For each brain model, the color bar is scaled to the maximal negative response, corresponding to the right edge of the bar. The yellow stars indicate the stimulation electrodes. Electrode numbers are shown at grid corners.

**Figure 3:**
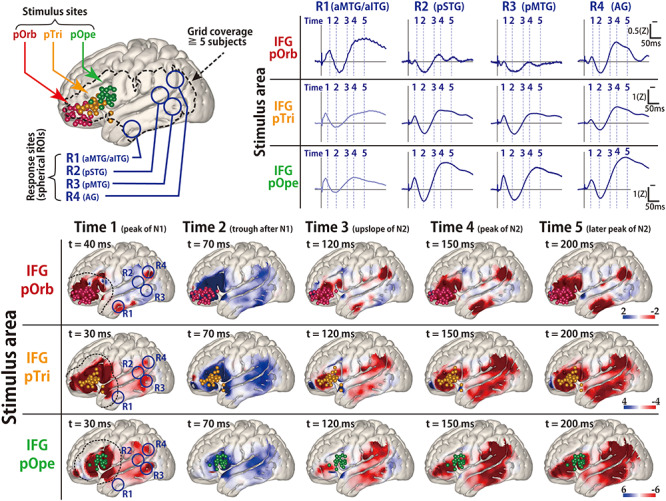
The average response map for the temporal lobe in all patients. (Upper left) Shown are all stimulus sites in the IFG transferred to MNI space stratified by subregion and labeled with colored spheres (red, pOrb; yellow, pTri; green, pOpe). The dotted contour indicates a recording area covered by data from no fewer than five patients. Any voxel within the dotted contour contains the recording electrodes from no fewer than five patients within 15 mm of the center of the voxel. (Lower) The table shows the averaged response maps in the time sequence stratified by IFG subdivision. The five time points were set to the peak of N1, the trough after N1, the upslope of N2, the peak of N2, and the peak of the latest N2 (when R1 in the pOrb stimulation and R4 in the pOpe stimulation are at their peak). The 4D versions are provided as a movie in the Supplementary Materials to facilitate an intuitive understanding of the response dynamics. While stimulation of pOpe elicited a prominent response broadly in the temporoparietal area, stimulation of pOrb elicited the response in the anterior part of the MTG and inferior temporal gyrus (ITG) and in the AG. Stimulation of pTri elicited a pattern that was a mixture of the patterns for the two stimulus areas. The dotted lines in the leftmost panels (Time 1) indicate the neighborhood of the stimulus sites (within 25 mm from each stimulus site). In these areas, the response is easily masked with the volume conduction of the large response at and around the stimulus site. (Upper right) The time course of the response amplitudes in the four ROIs. The radius of each ROI was 10 mm. We averaged the voxels inside each ROI to obtain the waveform. The four ROIs were located as follows: R1, in the anterior parts of the middle and inferior temporal gyri (aMTG/aITG); R2, in the posterior part of the superior temporal gyrus (pSTG); R3, in the posterior part of the MTG (pMTG); and R4, in the AG. The vertical lines indicate the timing of the averaged response map. The R1 waveforms in the pOpe and pTri rows are grayed out, indicating that they were hard to evaluate because of contamination by volume conduction. (We determined the contamination only when their time courses were similar to that of the stimulation neighborhood. Supplementary Figures 3 and 4 and the Supplementary Movie demonstrate that the electrodes around the stimulus sites elicit similar waveforms.) Color bars show the relative amplitude. Note that the average response map originally featured a *Z*-value for the amplitude but it was smoothed by a Gaussian kernel (FWHM 10 mm) for visualization. The Gaussian smoothing blurs the values across the adjacent voxels while keeping the sum, so the values decrease after smoothing.

## Results

### Visualization of Distinct Connectivity Patterns from IFG Subdivisions

The CCEP connectivity pattern varied distinctly when stimulation was administered through different subdivisions of the IFG. As shown in a representative case (Fig. 2), the distribution of the CCEP response changed depending on whether the stimulation was applied through the pOpe, pTri, or pOrb. In each patient, we observed distinct connectivity patterns for different IFG subdivisions. However, it is difficult to deduce a general rule of connectivity directly from individual cases due to limitations and variations in electrode coverage peculiar to each subject. Therefore, to systematically visualize the CCEP connectivity, we combined all the patient data into a standardized map of connectivity between the IFG and the lateral temporoparietal cortices.

Figure 3 shows the averaged response map obtained by stimulation of the three subdivisions in the IFG. The 4D CCEP movie (provided as Supplementary Materials) demonstrates the time course of the CCEP amplitude distribution. The waveforms in Figure 3 represent the averaged temporal dynamics of the voxels included in each region of interest (ROI). The center of the four spherical ROIs was located at representative N1 (early negative) response areas in IFG stimulation: “R1” was set on the N1 response area of pOrb stimulation; “R2” and “R3” on that of pOpe and pTri, respectively; and “R4” on the N1 response area common to all of the three stimulation areas. Note that ROI was not assigned in the neighborhood of the IFG (such as the anterior or middle part of STG) since the CCEP cannot be quantified accurately in the neighborhood of the stimulus site. It is hard to investigate the cortico-cortical connectivity in those areas by CCEP because the response is easily masked with the volume conduction of the large response at and around the stimulus site. This phenomenon is particularly prominent around the anterior portion of the Sylvian fissure where larger CSF volume is underneath the grid. (See Supplementary Figure 4 for the ROI analysis near the stimulus sites.) As the movie and the waveforms show, pOpe stimulation elicited prominent N1 responses in the posterior part of the temporal lobe (STG and middle temporal gyrus [MTG]) and adjacent parietal areas (supramarginal gyrus [SMG] and angular gyrus [AG]) around 30 ms after stimulus onset. After the N1 response, a larger and broader negative response (N2) with a peak latency of 150–200 ms was evoked in each response area (R2, R3, and R4). Although the averaged waveform suggests that the inferior part of the anterior lateral temporal lobe (R1) exhibited an N1 response for pOpe stimulation, we regarded this activity as a far-field potential reflecting the large response around the stimulus site; the R1 response shares the temporal dynamics of those electrodes around the stimulus site in the early phase (<20 ms), as demonstrated in the 4D CCEP movie. In contrast, pOrb stimulation elicited an N1 response in the anterior part of the ITG and MTG at around 40 ms after stimulation, which was followed by a larger N2 response in the same area (see the averaged waveform of R1 in the lower panel of Fig. 3). It also elicited an N1 response in the ventral part of the AG, which was followed by a large N2 response in the same area (see the averaged waveform of R4 in the lower panel of Fig. 3). pTri stimulation showed a response pattern intermediate between those of pOpe and pOrb stimulation because its CCEP response locations comprised the posterior STG, the posterior MTG, and the AG (N1 and N2), which resemble those of pOpe stimulation, and the anterior MTG and ITG (N2), which resemble those of pOrb stimulation.

### Topographical Analysis of Frontotemporal Connectivity

**Figure 4:**
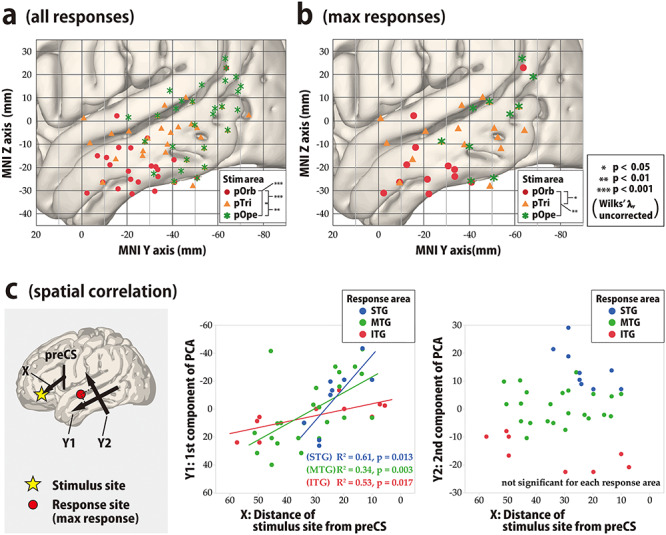
Topographical analysis of CCEP connectivity, temporal lobe. (*a*,*b*) To visualize the spatial distribution of the CCEP response in the lateral temporal cortices in response to stimulation in the IFG, we plotted the N1 response sites in the MNI *y*–*z* plane with different colors for each stimulus area in the IFG. All response sites that satisfied the amplitude criterion of >6 SD of the baseline fluctuation were plotted. (*a*) Shown is the spatial distribution of all response sites in the lateral temporal area in the MNI *y*–*z* plane. The red circles, orange triangles, and green asterisks indicate responses to stimulation in pOrb, pTri, and pOpe, respectively. We tested the difference in response distribution for each pairwise combination of the three stimulus groups (pOrb, pTri, and pOpe) statistically by the *F*-test. We selected the stimulus sites so that the three stimulus groups would be mutually exclusive, excluding those stimulus sites with electrodes located over the different subdivisions of the IFG. Please note that all the significant CCEP responses (>6 SD of the baseline activity) are plotted here, including those with small amplitude, which would highlight smaller responses clearer than in Figure 3 (amplitude-based coloring). (*b*) Shown is the spatial distribution of the maximal sites. The legend for the symbols is the same as in (a). The differences among the 2D distributions were tested with Wilks’ lambda as above. (*c*) We generated two scatter plots to show the spatial correlation between X and Y1 and between X and Y2. In these scatter plots, linear regression analysis for each response area (STG, MTG, and ITG) was performed. Shown is the spatial correlation between the stimulus sites and the maximal response sites. The locations of the IFG stimulus sites are approximated as their MNI coordinates on the anterior–posterior axis (i.e., the distance from the midpoint of the ventral third of the precentral sulcus, PreCS). PCA shows that the main axis of the response distribution is the anterior–posterior axis of the temporal lobe (Y1, first PCA component, left panel). Y1 is linearly correlated with the anterior–posterior position of the stimulus sites in every part of the temporal area, i.e., in the superior temporal gyrus (STG), MTG, and ITG (middle panel). Note that the value is always positive in the IFG and that a larger value indicates a more anterior location. We also used the second PCA component as the Y2-axis, which is orthogonal to the Y1-axis. Larger values on the Y2-axis indicate more superior locations. Y2 values are not correlated with the location of the stimulus site (right panel).

To validate the differences in connectivity pattern visualized with the 3D average response map statistically, we investigated the topographical distribution of the early negative (N1) responses (Fig. 4*a*,*b*). In pOrb stimulation, the N1 response sites clustered in the anterior inferior part of the lateral temporal area. However, in pOpe stimulation, the N1 response sites clustered in the posterior part of the lateral temporal area. Stimulation of pTri elicited N1 responses at sites between the former two clusters. The spatial distribution was significantly different between any pair of the three parts, pOrb, pTri, and pOpe, by Wilk’s lambda (*P* < 0.01). When the scatter plot was confined to the max response sites for N1, the differences remained statistically significant (Fig. 4*b*).

The finding that pTri stimulation showed a connectivity pattern intermediate between those of pOpe and pOrb stimulation implied a gradient in the connectivity pattern revealed by IFG stimulation. We performed a linear regression analysis based on the coordinates of stimulus sites and response sites. The locations of the stimulus sites were linearly correlated with those of the N1 max response sites in the lateral temporal area (Fig. 4*c*). In the anterior–posterior axis, the regression line could be calculated both in each temporal gyrus (STG, *Y* = 2.37 × *X* − 56.27, *R*^2^ = 0.61, *P* = 0.013; MTG, *Y* = 1.12 × *X* − 28.77, *R*^2^ = 0.34, *P* = 0.003; and ITG, *Y* = 0.43 × *X* − 3.59, *R*^2^ = 0.53, *P* = 0.017) and in the whole temporal cortex (*Y* = 0.88 × *X* − 20.29, *R*^2^ = 0.32, *P* < 0.0001). However, no significant correlation was observed in the superior–inferior axis. In summary, the more anterior part of the IFG connects to the more anterior part of the lateral temporal area while the more posterior IFG connects to the more posterior temporal area, indicating a connectivity gradient along the anterior–posterior axis. We also analyzed all response sites instead of the maximal response sites and observed a similar gradient of connectivity supported by linear regression analyses.

### Fronto-Parietal Connectivity

**Figure 5:**
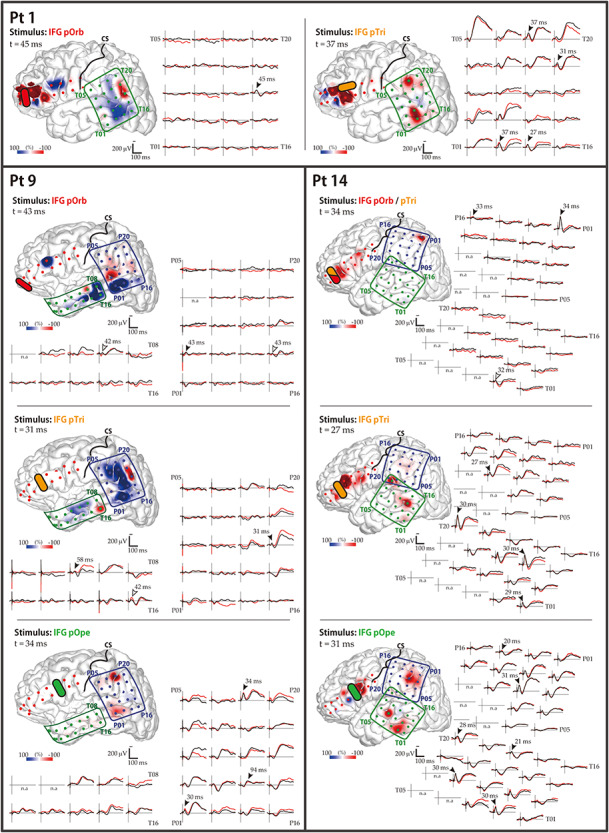
CCEP responses to IFG stimulation: parietal lobe. Shown are the data for the three patients (1, 9, and 14) who showed frontoparietal connectivity. The waveforms in the temporal and parietal grids are shown. The black and red waveforms show the average evoked potential from two consecutive sessions. The black arrowheads indicate the maximal N1 response sites automatically extracted from the CCEP database. The white arrowheads indicate the maximal N1 sites added by visual inspection, which were missed by the automatic algorithm due to poor reproducibility. The brain surface models show the spatial distribution of the early-phase response amplitudes, measured at the peak time of the parietal N1 response. The color bar is scaled to the maximal amplitudes of the parietal N1 responses in each panel. Yellow bars indicate the stimulation electrode pairs; “CS” and black lines, the central sulcus. Electrode numbers are shown at grid corners.

The averaged response map clearly demonstrated the connectivity between each IFG subdivision and the inferior parietal area. In the present study, pOrb stimulation elicited discrete parietal CCEP responses in three of five patients who had a grid on the parietal lobe (Fig. 5). In all three patients, pOrb stimulation elicited an early negative (N1) response in the AG, while pTri or pOpe stimulation elicited an N1 response in the SMG. The N1 peak latency in the inferior parietal area was always longer in pOrb stimulation than in pTri and pOpe stimulation, as shown by the following data: patient 1, 45 ms (pOrb stimulation, AG) versus 31 ms (pTri stimulation, SMG) and 37 ms (pTri stimulation, SMG); patient 9, 43 ms (pOrb stimulation, AG) versus 31 ms (pTri stimulation, AG) and 34 ms (pOpe stimulation, SMG); patient 14, 34 ms (pOrb stimulation, AG) versus 27 ms (pTri stimulation, SMG), and 31 ms (pOpe stimulation, SMG).

### Reciprocality

We investigated the rates of occurrence of reciprocality in the connections between the IFG pOrb and the temporal and parietal areas (Table 2). We stimulated pOrb through a total of 41 stimulus sites and observed an F→T connectivity in 52 electrodes. Due to limitations of time in the operating room, we were able to stimulate only 36 electrode pairs that included at least one of the F→T response sites and observed 25 reciprocal T→F connections (25/36, 69.44%) with max responses at the “initial” stimulus site. When the analysis was confined to max F→T response sites (22 sites), we were able to stimulate 18 electrode pairs that included at least one of the max response sites and observed 13 reciprocal T→F connections (13/18, 72.22%). We performed a similar analysis for the no-response electrodes as a negative control. We aggregated all CCEP recordings that included at least one no-response electrode (333 total); among them we found 30 reciprocal connections (30/333, 9.01%). The rate of occurrence of reciprocal connections was significantly higher at max response sites and at all response sites than at no-response sites (unpaired *t*-test, *P* < 0.0001, uncorrected).

**Table 2:** The occurrence rates of reciprocality in connectivity from IFG pOrb

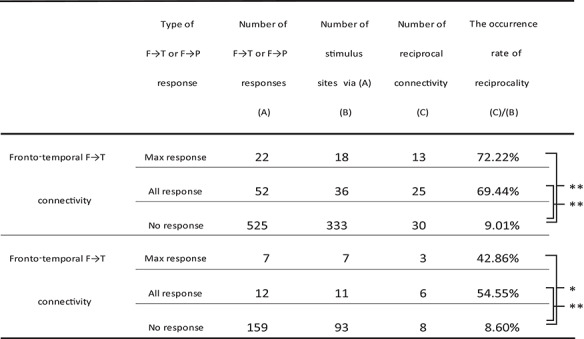

Note: The rate of occurrence of reciprocality was defined as the division of the number of the reciprocal (T→F or P→F) connectivity by the number of the stimulus sites via the temporal or parietal response sites as at least one of the paired stimulus electrodes. The reciprocality rate was calculated for the F→T and F→P connectivity separately. We performed similar analysis for the three groups stratified by the type of F→T or F→P response; max response, all response, and no response. See the Supplementary Material for details of the method used to judge reciprocality.

^*^ *P*-value < 0.05 (unpaired *t*-test, uncorrected).

^**^ *P*-value < 0.0001 (unpaired *t*-test, uncorrected).

**Table 3:** N1 latency of the CCEP response

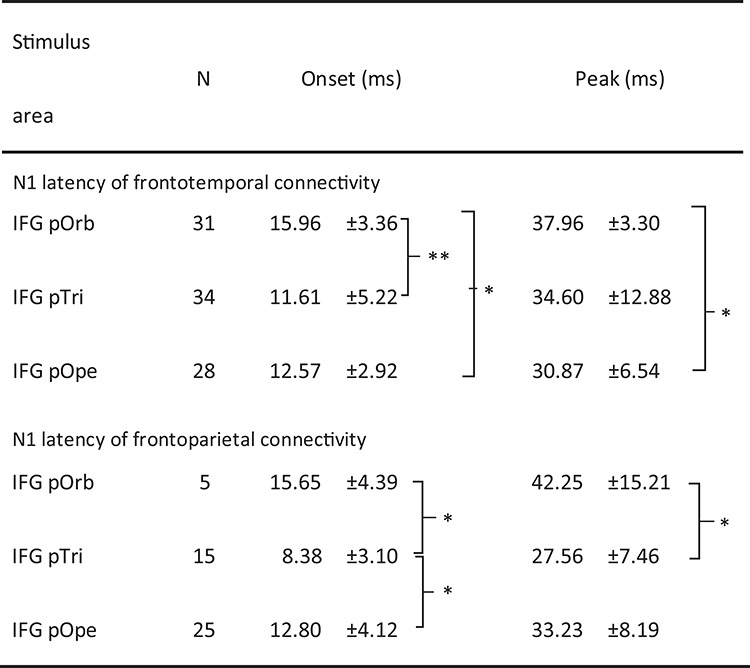

^*^ *P*-value < 0.05 (unpaired *t*-test, Bonferroni corrected).

^**^ *P*-value < 0.001 (unpaired *t*-test, Bonferroni corrected).

We performed a similar investigation of the connectivity between pOrb and the parietal area and observed similar results, although the numbers were smaller: 3 reciprocal P→F responses upon stimulation of 7 max response sites (3/7, 42.86%) in the F→P connections, 6 reciprocal responses upon stimulation of the 11 response sites (6/11, 54.55%), and 8 reciprocal responses upon stimulation of 93 no-response sites (8/93, 8.60%). The occurrence rate was significantly higher at both the maximal response sites and all response sites than at no-response sites (unpaired *t*-test, *P* < 0.05, uncorrected).

### Latency and Estimated Conduction Velocity

Table 3 shows the onset times and peak latencies of all measured waveforms. In the lateral temporal cortices, the N1 onset latency was significantly longer with pOrb stimulation than with pTri or pOpe stimulation (unpaired *t*-test, *P* < 0.005, uncorrected). The peak latency was longer with pOrb stimulation than with pOpe stimulation (unpaired *t*-test, *P* < 0.005). Similarly, in the inferior parietal lobule, pOrb stimulation showed longer latencies at onset and peak than did the other two subdivisions, although the number of available pOrb stimulations was small (*n* = 5).

We plotted the onset latency versus the Euclidean distance between the stimulus and response sites to investigate conduction velocity (see Supplementary Figure 2). The slope of the regression line (*Y* = 0.076 × *X* + 8.2) indicated an approximate conduction velocity of 13.2 m/s (*P* < 0.05), with large variability (*R*^2^ = 0.047). We also created scatter plots for each stimulus area (pOrb, pTri, and pOpe) to compare the conduction velocity between these areas, but no significant regression lines were found.

### Comparison with Resting-State Functional Connectivity

**Figure 6:**
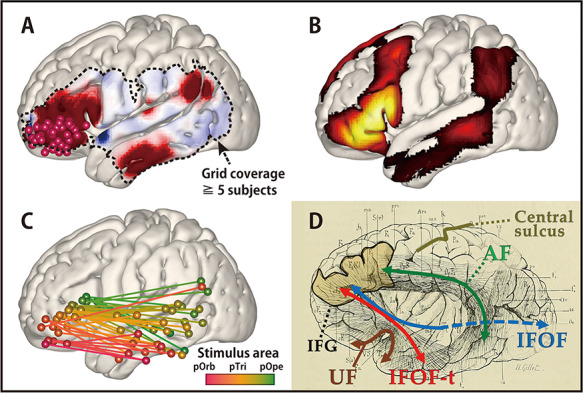
Comparison of connectivity values discovered by CCEP and rs-fMRI: A connectivity gradient. (*A*) The averaged response map produced by stimulating pOrb, delayed phase (same as in Fig. 3). (*B*) The functional connectivity from pOrb derived from the NeuroSynth database. In this study, the seed voxels were decided as the pOrb stimulus sites. The connectivity pattern in the lateral temporo-parietal area resembles that of CCEP. (*C*) The connectivity gradient between the IFG and MTG as assessed using the CCEP database. The data used in this figure are the same as that in Figure 4, although this figure shows both the stimulus and response sites in the MNI *y*–*z* plane. The color gradation indicates the anterior–posterior coordinate of the stimulus sites. The gradation from red to green corresponds to the transition from the anterior to the posterior stimulation site. All pairs of stimulus and max response sites are plotted in the MNI *y*–*z* plane to illustrate the connectivity gradient. (*D*) An illustration of the long tracts in the vicinity of the IFG, overlaid on the reprinted schema of white matter dissection from the classical textbook “Anatomie des centres nerveux” (Dejerine and Dejerine-Klumpke 1895). Major pathways are annotated with colored arrows, the IFG is outlined with a black line, and the central sulcus is outlined with a beige line. A fan-shaped structure can be seen connecting the frontal lobe and the ATL through the temporal stem.

The connectivity pattern elicited by stimulation of pOrb was generally similar to that of the resting-state functional connectivity obtained from the NeuroSynth database by specifying the seed as a stimulus site in IFG pOrb (Fig. 6*A*,*B*). The distribution was similar between the two connectivity modalities in the anterior part of the ITG and MTG and in the inferior parietal lobule; however, a difference was observed in the posterior part of the MTG (rs-fMRI positive, CCEP negative). The discrepancy is attributable to the presence of an indirect correlation via the posterior IFG (pTri and pOpe) for rs-fMRI connectivity because pTri showed a strong correlation with both pOrb and the posterior MTG. Because the resting-state functional connectivity is a measure of correlation, it inevitably visualizes a chain of strong relationships as a single indirect relationship.

## Discussion

Based on a compilation of CCEP data, we investigated the connectivity pattern between the IFG and the temporoparietal area. The CCEP response pattern indicated a gradual transition of connectivity from stimulus sites in the posterior IFG (pOpe) to those in the anterior IFG (pOrb). Topographical analysis of the stimulus and response sites confirmed the presence of a connectivity gradient between the IFG and the temporal lobe along the anterior–posterior axis. In particular, the anterior part of the IFG (pOrb) showed connectivity to the anterior lateral temporal area, which has not been well delineated by frozen dissection, although a recent study utilizing probabilistic tractography demonstrated the connectivity between the pOrb and the lateral surface of the rostral temporal lobe (Binney et al. 2012). We discuss the functional and clinical aspects of these results below.

### Candidate White Matter Pathways Between the Anterior IFG and Temporal Lobe

The present CCEP findings revealed connections between the anterior IFG (pOrb) and the anterior lateral temporal lobe. Although CCEPs do not provide direct evidence about the underlying white matter pathways, recent in vivo and postmortem anatomical studies utilizing diffusion tractography and frozen dissection (Klinger’s method) potentially yield some clarification of the white matter fibers terminating in the IFG. They consistently found that the anterior part (pOrb) and posterior part (pTri and pOpe) of the IFG receive distinct fibers. That is, pOrb receives the termination of the IFOF (especially the superficial component) and UF, while pTri and pOpe receive terminations from the SLF and AF (Catani et al. 2005; Barrick et al. 2007; Glasser and Rilling 2008; Lawes et al. 2008; Vassal et al. 2016). Based on these anatomical findings, it seems plausible that, upon pOrb stimulation, the electrical impulse is conveyed through the IFOF or UF rather than through the SLF or AF to the anterior lateral temporal lobe. A different connectivity pattern from each IFG subdivision has also been indicated by probabilistic tractography (Anwander et al. 2007). That study demonstrated that the connectivity signature originating from pOpe represented the AF while that from pOrb represented the UF and IFOF. Given that both structures pass through the extreme capsule in the temporal stem, we expect that a pathway exists between the IFG pOrb and the anterior lateral temporal lobe via the extreme capsule. Since the UF terminates mainly in the temporal pole, which was not covered by the electrode grids in this study, the connectivity between the IFG pOrb and the anterior lateral temporal lobe implies the existence of a temporal branch of the IFOF, which we referred to as IFOF-t. The bundle comprised of the UF and IFOF-t can be depicted as a fan-shaped structure spreading over the temporal lobe, as illustrated in the classical textbook by Dejerine and Dejerine-Klumpke (Fig. 6*D*). Here, we call this structure the “frontotemporal radiation.”

Although the IFOF-t has not been found in recent frozen dissection or tractography studies, the existence of such connectivity is supported by our finding of reciprocality for this connectivity (Table 2) and by similarity with the resting-state functional connectivity (Fig. 6). The reciprocal connectivity under discussion implies the functional relevance of the connectivity. The resemblance between CCEP connectivity and rs-fMRI connectivity validates the existence of connectivity, as the CCEP amplitude is reported to correlate with the rs-fMRI connectivity (Keller et al*.* 2014a). Furthermore, in one recent study, where whole-brain deterministic tractography was performed and virtual dissection of the UF and IFOF by a novel “stem-based” approach was carried out, a fanning structure comprising the UF and IFOF was visualized, including what we call IFOF-t (Hau et al. 2016a). There are two reasons for the discrepancy between the CCEP and the frozen-dissection/tractography results. One is the presence of small fiber diameters in the ventral pathway (UF and IFOF) as revealed in an electron microscopic investigation (Liewald et al. 2014). The other is that the IFOF-t is closely bundled with many other major long tracts like all fibers running through the extreme capsule complex and temporal stem (Martino et al. 2010b; Peltier et al. 2010; Ribas et al. 2015; Bajada et al. 2017a). When using the frozen dissection technique, the frozen white matter is peeled along the principal fiber direction, which means that small fibers running across the major direction are destroyed (Zemmoura et al. 2016). The tractography technique is based on the direction of local water diffusivity, which represents the principal direction of fibers within the voxel and will, therefore, neglect the small crossing fibers (Tuch et al. 2003; Mukherjee et al. 2008). CCEP relies on neurophysiological measurement, which makes it a more sensitive method for tracing crossing fibers.

### Candidate White Matter Pathways Between the Anterior IFG and Parietal Lobe

In the present study, pOrb stimulation elicited discrete responses in the parietal area and stimulation at the response sites revealed reciprocal connections, though we need a larger number of patients to verify the results. Based on the above discussion, pOrb stimulation is assumed to propagate through the IFOF. In the parietal termination, CCEP responses were found predominantly in the AG, which is known to be one of the posterior terminations of the IFOF (Caverzasi et al. 2014; Hau et al. 2016b). We cannot exclude the possibility that anterior IFG-AG connectivity is mediated by the SLF III because this tract is also reported to project to the AG and anteriorly as far as the dorsolateral prefrontal cortex (Mars et al. 2011; Seghier 2013; Parlatini et al. 2017). However, to the best of our knowledge, there has been no report confirming that the frontal termination of the SLF III clearly includes the IFG pOrb.

### Longer Latencies of CCEP with Stimulation in pOrb

The latency of the CCEP response by the stimulation of pTri or pOpe was consistent with the previous reports (Matsumoto et al. 2004; Yamao et al*.* 2014, 2017). In contrast, the relatively long latency (peak around 40 ms) of CCEP responses was seen with pOrb stimulation. This is consistent with the existence of the IFOF-t because the fiber diameter of the ventral pathway (UF and IFOF) was found to be smaller than that of the dorsal pathway (SLF) in an electron microscopic investigation (Liewald et al. 2014); furthermore, conduction velocity is well known to increase linearly with fiber diameter (Hursh 1939). The longer response latency seen with pOrb stimulation is also consistent with the finding that pOrb showed a lower myelin density than pTri or pOpe in recent myelin-density mapping studies (Glasser and Van Essen 2011; Glasser et al. 2016). The observation of a lower myelin density supports the possibility that the axons originating in the area are less myelinated, and therefore have lower conduction velocities than those from pTri or pOpe.

### A Connectivity Gradient in the IFG

The linear regression analysis based on the coordinates of the stimulus and response sites indicated that the IFG is connected to the lateral temporal cortex with a gradation in the anterior–posterior axis. This is not only consistent with the presence of a fan-shaped structure but also implies a seamless transition from the dorsal stream to the ventral stream in the IFG. Recently, a functional and connectivity gradient along the anterior–posterior axis was found not only in the IFG (Hagoort 2005; Xiang et al. 2010; Udden and Bahlmann 2012; Thiebaut de Schotten et al. 2017) but also in the temporal lobe (Bajada et al. 2017b; Jackson et al. 2018). Interestingly, at both sites, the anterior part was associated with a modality-general network and the posterior part with a modality-specific network. As Figure 6*C* shows, our results support graded functional differentiation in the IFG. Although the concept of a connectivity gradient has been mentioned previously in the literature, this is the first report of an anterior–posterior gradient in the temporal projection from the IFG based on an electrical tracing method. Here, again, the gradual nature of CCEP connectivity agrees with the fanning structure illustrated in an historical textbook because the fan-shaped bundle of lines was drawn not only in the anterior part but also in the posterior part of the IFG (Fig. 6*D*).

### Possibility of Parcellation Based on CCEP Connectivity

Our CCEP data indicate not only the existence of two functional networks in the IFG but also the possibility of parcellation based on CCEP connectivity. Although we can find numerous reports on connectivity-based parcellation of the human brain by means of diffusion tensor imaging (Anwander et al. 2007; Cloutman and Lambon Ralph 2012) and rs-fMRI (Arslan et al. 2018; Jackson et al. 2018; O’Muircheartaigh and Jbabdi 2018), to the best of our knowledge, there are no reports on that of CCEP connectivity, although there have been reports of a whole brain connectivity matrix (ROI analysis based on a template) collecting individual CCEP data (Entz et al. 2014; Donos et al. 2016). As we showed at the individual (Fig. 2) and group average (Fig. 3) levels, a 1-cm difference in the stimulus site resulted in a completely different connectivity pattern, and the average connectivity pattern was different for each stimulus area in the IFG. Compared with the MRI-based methods, which make use of whole brain connectivity data, CCEP-based parcellation seems to be difficult because the spatial resolution is no better than MRI and the CCEP data are collected only from beneath the implanted electrodes. However, the CCEP method is expected to surpass the MRI-based method in some clinical situations (such as peritumoral edema and arteriovenous shunt, which undermine the basic requirements concerning water diffusibility and neurovascular coupling, respectively) because it probes connectivity directly by electrical stimulation. In that sense, it is worth challenging to perform connectivity-based parcellation solely by the CCEP method. CCEP connectivity-based parcellation is clinically important for such an eloquent area as the IFG on the dominant side because it enables functional mapping without requiring the patient’s conscious cooperation, which is often unachievable in children or patients with cognitive disturbance. Although these results must be verified in a larger population, the present study confirms that parcellation of a functionally confluent area such as the IFG solely by CCEP is feasible.

### Functional Implications of the Connectivity Determined from pOrb

The present study demonstrated that visualizing the connectivity from the anterior part of the IFG to the anterior part of the MTG/ITG is feasible using CCEP methodology. As discussed above, both the frontotemporal and frontoparietal connectivity from pOrb are considered to be mediated by subcomponents of the IFOF. From a functional point of view, the IFOF is reported to have a semantic function, as evidenced by intraoperative electrical stimulation at subcortical white matter sites along the IFOF (Duffau 2005). IFOF-t also seems to be involved in a semantic function at its cortical terminations according to the following lines of evidence. The anterior IFG, which is the frontal termination of the IFOF-t, has been revealed by means of fMRI to engage in controlled semantic retrieval (Wagner et al. 2001; Krieger-Redwood et al. 2015), and transcranial magnetic stimulation in this area prolonged the response latency in a synonym judgment task (Gough et al*.* 2005; Hoffman et al*.* 2010). With regard to the cortical termination in the temporal lobe, a PET activation study in healthy subjects revealed involvement of the anterior MTG and ITG in comprehension of words presented auditorily and visually (Spitsyna et al*.* 2006), and studies using voxel-based lesion symptom mapping in aphasic patients found associations with semantic error in the lateral anterior temporal cortex (Walker et al. 2011). It is likely that the network between these two regions, namely the IFOF-t, has a role in semantic processing.

We also determined the connectivity between the pOrb and AG, although the number of patients was small (Fig. 5). As mentioned previously, tractography shows that the AG is connected with the pOrb via the parietal branch of the IFOF (Caverzasi et al*.* 2014; Hau et al*.* 2016b), which has been confirmed by frozen dissection (Curran 1909; Martino et al*.* 2010a). Like pOrb, the AG is associated with a semantic role according to a meta-analysis of neuroimaging studies focusing on semantic processing (Binder et al. 2009), although the behavior of AG during task fMRI is significantly different from that of ATL (Humphreys et al. 2015), which is the semantic representational hub, as evidenced by a transcranial magnetic stimulation study (Hoffman et al*.* 2010). The fact that both the pOrb and AG are associated with semantic processing implies that the parietal branch of the IFOF may be associated with semantic processing. Even when looking outside the semantic network, a direct connection between the pOrb and AG deserves attention because not only the AG but also the anterior IFG is involved in the default mode network (Buckner et al. 2008).

### Clinical Implications of the CCEP Examination in IFG

Previous reports from our group have shown that the intraoperative CCEP with stimulation of pTri and pOpe is clinically useful for probing the posterior language area (Wernicke’s area) through AF (Yamao et al*.* 2014). The present study extended this clinical implication to map the whole connectivity along the anterior–posterior axis of the IFG. The graded connectivity along the IFG and the temporal lobe underlies the functional gradient in both areas as discussed above; the more anterior region connects to the more modality-general and the more posterior to the more modality-specific. This comprehensive IFG connectivity mapping would allow delineation of the functional regions located in the anterior part of the IFG and the temporal lobes, such as the semantic control area. In the future, we could refer to the “4D CCEP map” to guide the location of electrode placement when more patients are enrolled to refine its quality for clinical use.

In this study, we could not map the cortical functions in the anterior part of the IFG and the temporal lobe during awake surgery. Specialized tasks for semantic cognition will be needed to investigate semantic function. Such deliberate tasks will be more time-consuming than intraoperative tasks such as picture naming, and will demand more attention and motivation from patients, which is difficult to achieve in an intraoperative setting. Mapping studies in patients with chronically implanted electrodes for epilepsy surgery will delineate more deliberate cognitive functions in these areas. We believe mapping and preserving these higher functions out of the classical “eloquent” area, i.e., Broca’s area, would improve quality of life in patients undergoing neurosurgery. In order to preserve the white matter pathway, including the temporal stem, sequential intraoperative CCEP evaluation would be clinically beneficial for patients who have lesions in the insula or temporal stem. Detailed longitudinal neuropsychological assessments of language and semantic function should be performed in patients undergoing neurosurgery involving the cortical and subcortical areas, given their functional relevance.

### Study Limitations

This study investigated and clearly illustrated the connectivity between the IFG and temporoparietal area. However, some limitations should be noted.

First, we could not fully exclude the pathological effect of a lesion, although we excluded patients who had lesions or massive edema around the temporal stem, which is the key structure in the ventral language network.

Second, the location of the electrodes was determined by the clinical requirements for monitoring language function and by safety issues. For example, electrode grids are placed on a flat surface for stability and for keeping a distance from bridging vessels for safety. To compare the connectivity patterns among all the three subdivisions in the IFG, we included only those patients in whom all three IFG subdivisions were covered by electrodes.

Third, there was no direct evidence in this study of the white matter underpinning connectivity between the anterior IFG and anterior MTG/ITG. If we observe an evoked potential in both terminals (the pOrb and the anterior MTG/ITG) by single-pulse electrical stimulation of the white matter, it would provide proof of underpinning. We indeed attempted to stimulate the IFOF on the superior wall of the inferior horn through the removal cavity using a 1 × 4 strip electrode after anterior temporal lobectomy. However, the result was widespread CCEP responses in almost all frontal and temporal electrodes, which made interpretation difficult (unpublished data). At present, we have no direct evidence although a smaller electrode and a weaker stimulus intensity may improve the situation.

Fourth, this study includes no functional mapping of connectivity, as mentioned in the previous section. Further studies are needed to assess the function of the connectivity observed here using electrical stimulation of the white matter and cortices in both terminals. These assessments should be included in future studies because we believe that it is necessary for future neurosurgeons to be aware of the functions of the neural structures within the operative field even if they are out of the classical “eloquent” area.

Fifth, the number of patients included in the study is smaller than in other studies that visualized connectivity using CCEP. Recently, several connectivity maps based on a larger population of patients with implanted electrodes have been published (Entz et al. 2014; Donos et al*.* 2016; Trebaul et al. 2018). Although our study includes a relatively small number of patients, it is noteworthy that all our patients underwent single-pulse stimulation in all subdivisions of the IFG available for observation of differences in connectivity patterns. Furthermore, all the data were collected from one institution, which eliminates concerns about differences in stimulus parameters and variations in the measurement environment.

Finally, various methods other than CCEP can be used to probe cortico-cortical connectivity, such as diffusion tensor imaging, rs-fMRI, or anatomical tracer studies, although no one method provides a complete account by itself. To overcome this problem, integration of the CCEP data with the findings from other modalities should be targeted in a future study. In the present study, we compared the CCEP connectivity with the rs-fMRI at the group level (Fig. 6). Future studies incorporating the multimodal evaluations at the individual level warrant comprehensive understanding of the human connectome.

## Conclusion

Our intraoperative CCEP data showed that the anterior IFG is connected to the anterior MTG/ITG. Combined with prior anatomical knowledge about the frontal termination of language-related fibers, these findings confirm that the anterior IFG has a connection with the anterior MTG/ITG through the ventral stream (referred to herein as the IFOF-t), which appears as a fan-shaped structure, known here as the “frontotemporal radiation,” together with the UF and the classical IFOF. The anterior–posterior gradient in connectivity observed between the IFG and temporal area suggests the presence of a gradual transition in IFG efferents between the ventral and dorsal streams.

## Funding

Ministry of Education, Culture, Sports, Science, and Technology (grant numbers 15H05874, 17H05907); Japan Society for the Promotion of Science (grant numbers 17K10892, 18H02709, 18K19514, 19K17033, 19K18424); Medical Research Council, UK (MR/R023883/1 to M.A.L.R.).

## Notes

We thank Drs Tamaki Kobayashi, Taku Inada, Yuki Takahashi, Sei Nishida, and Rika Inano for their cooperation with the intraoperative CCEP examinations.

*Conflict of Interest*: The Department of Epilepsy, Movement Disorders, and Physiology, Kyoto University Graduate School of Medicine conducts Industry-Academia Collaboration Courses, supported by Eisai Co., Ltd, Nihon Kohden Corporation, Otsuka Pharmaceutical Co., and UCB Japan Co., Ltd.

## Supplementary Material

Supplementary_Materials_bhaa065

Supplementary_Movie_bhaa065
