# Supplementary material for: Connectivity Gradient in the Human Left Inferior Frontal Gyrus: Intraoperative Cortico-Cortical Evoked Potential Study
Source: Cereb Cortex. 2020 Mar 31;30(8):4633–50. doi: 10.1093/cercor/bhaa065 (PMC7325718; doi:10.1093/cercor/bhaa065)
Supplement: Supplementary_Materials_bhaa065 [file supplementary_materials_bhaa065.docx]

**Figure S1. Scatter plot of CCEP latency.** The onset latency (on X axis) and the peak latency (on Y axis) are plotted about all remote CCEP responses in this study (N = 1626). Note that N1 and N2 responses are both included separately even if they are found in the same waveform. The color of dot indicates the classification suggested by the cluster analysis (Ward’s method). The red dots are regarded as typical N1 responses. The blue dots are typical as N2. The green dots seems to constitute another entity between N1 and N2. The bar graphs on the top and right of the scatter plot show the histogram of onset and peak. The red bars in the histogram corresponds to the cluster of typical N1 response.


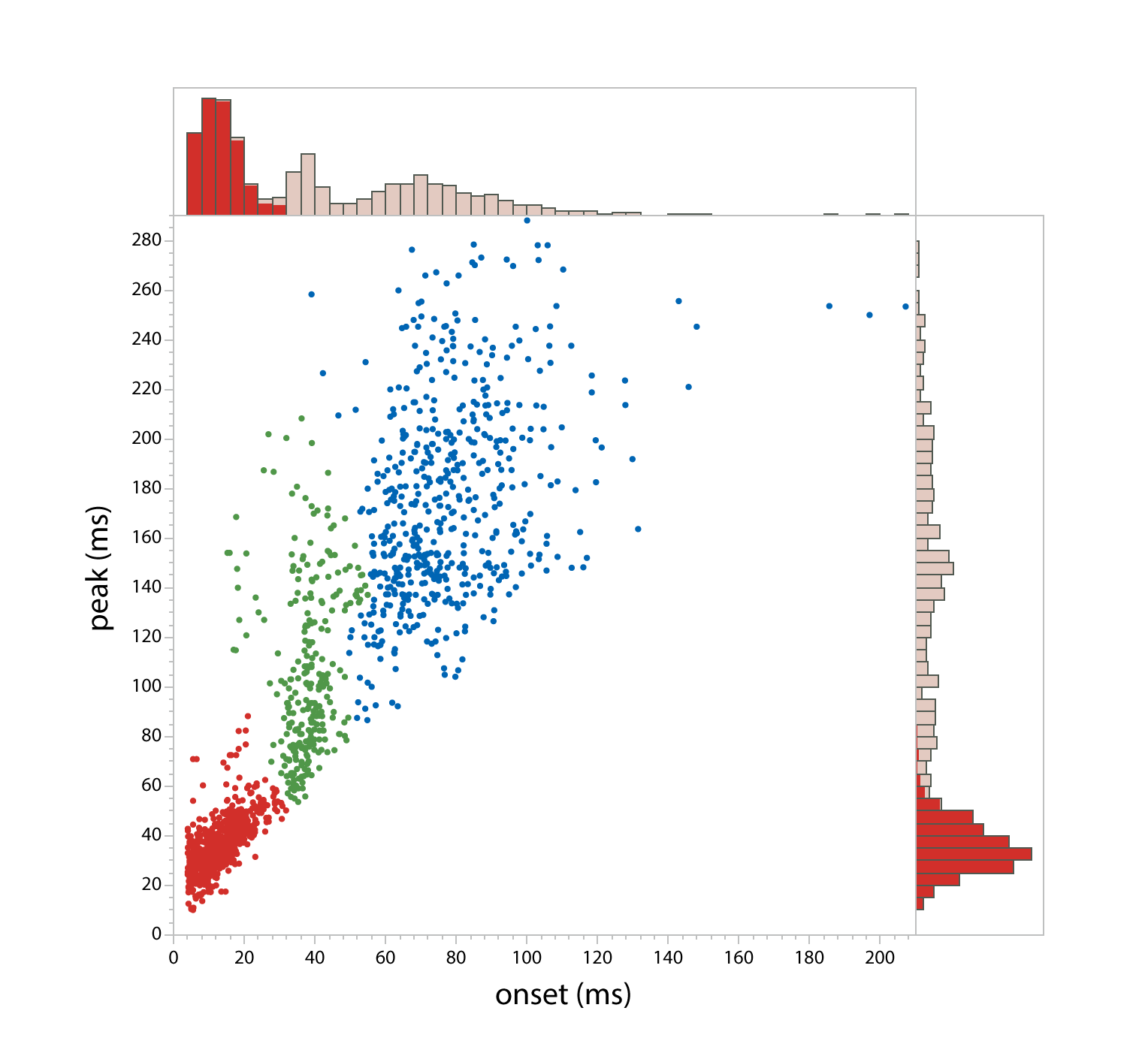


**Figure S2. Scatter plot of the stimulus-response distance and the onset latency of the N1 response.** All temporal responses by the stimulation of IFG (N = 93) are included. Red line indicates the regression line for the stimulation of all parts in IFG (p < 0.05). No regression line could be drawn for each part in IFG.


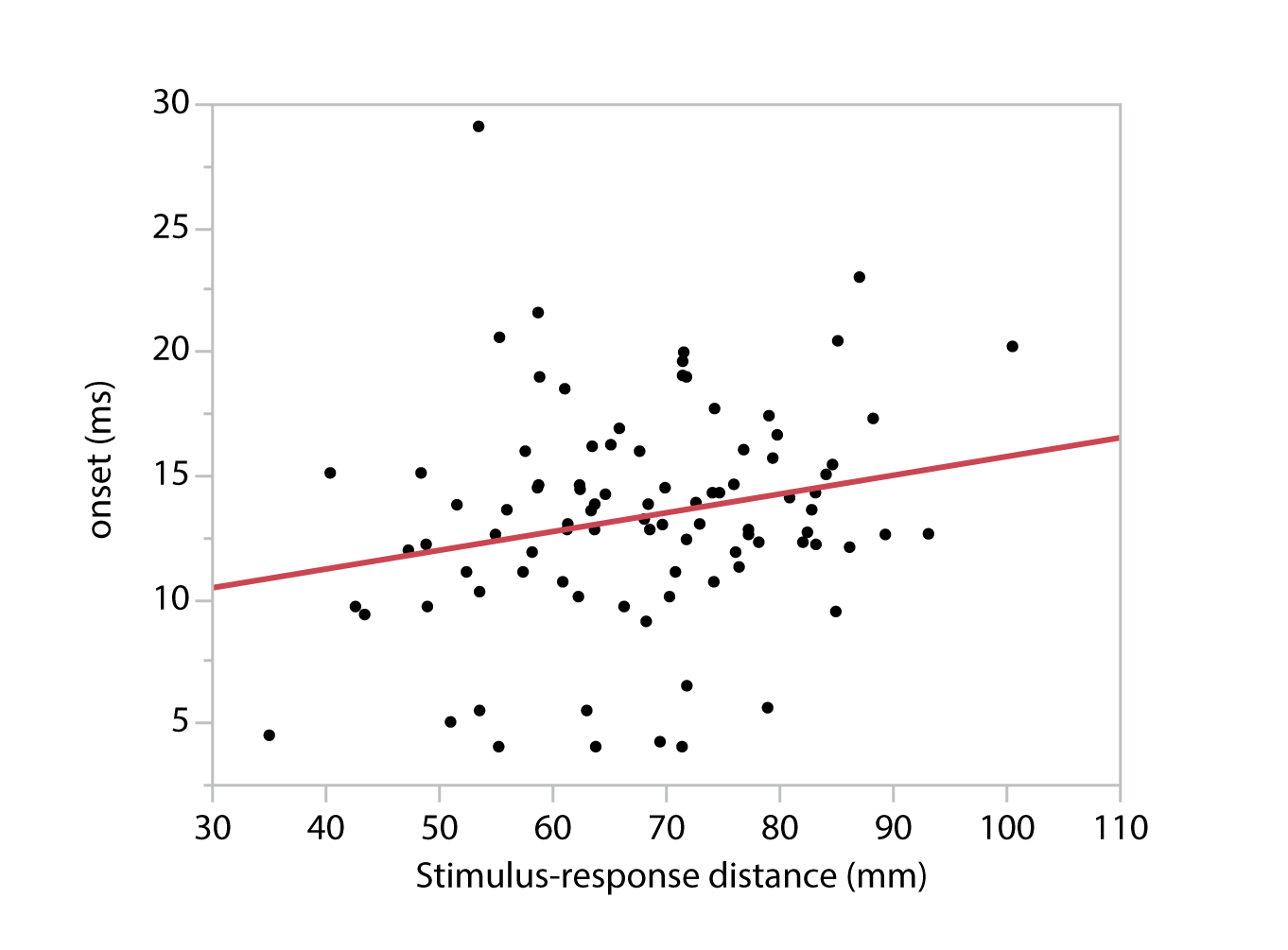


**Figure S3. Volume conducted response in individual level.** We selected Pt 6 (patient no. 6) as a representative case showing a volume conduction phenomenon by IFG pTri stimulation in the anterior temporal cortex. The brain model in the left lower panel indicates the location of electrodes. The stimulus site was indicated by an orange bar. Red vertical lines were drawn to indicate the timing of N1 peak at T20, demonstrating that several electrodes in the anterior temporal cortex elicited waveforms in a similar shape. Red dots indicates the volume conducted responses suspected by waveform.


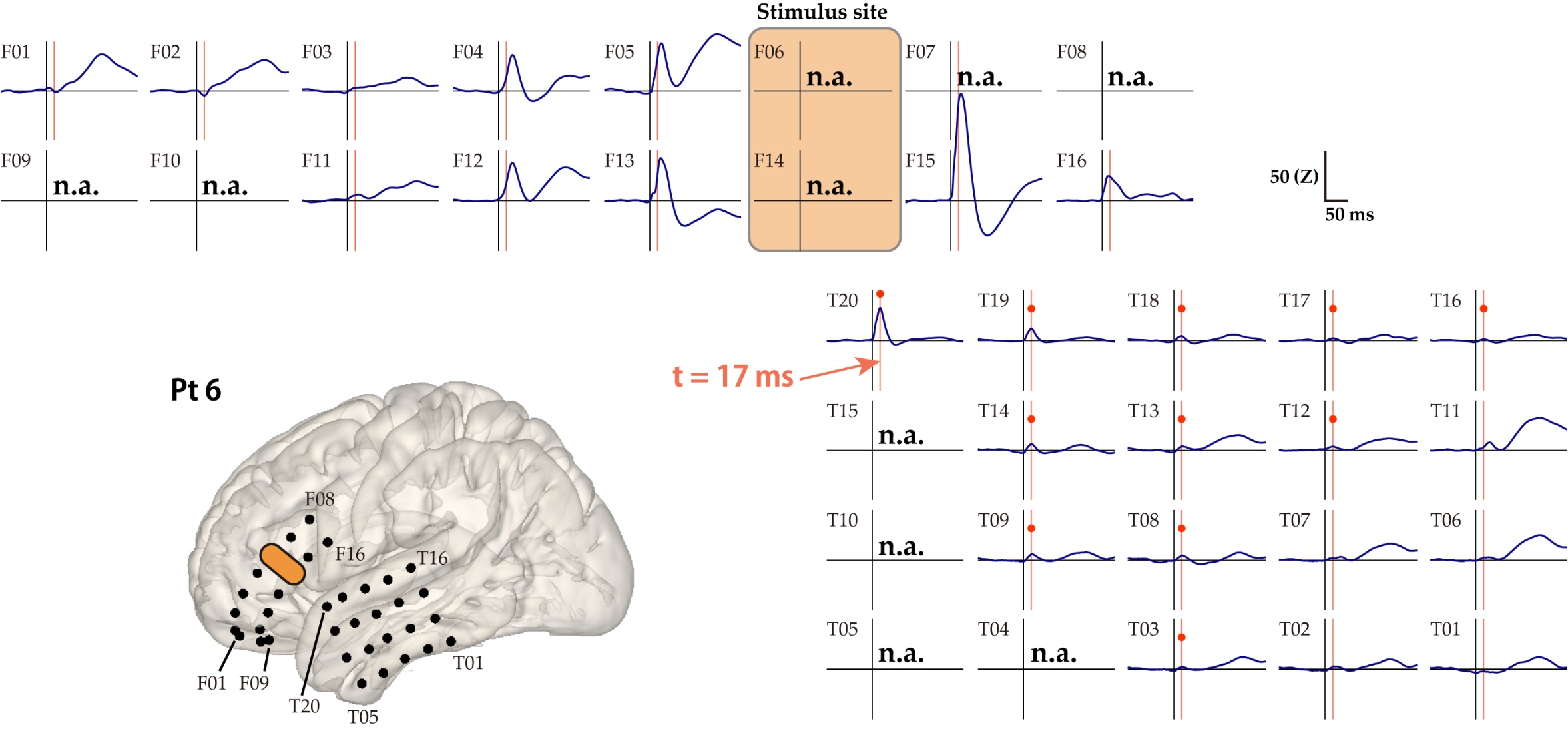


**Figure S4. The ROI analysis in the all-patients average response map focusing on the volume conduction phenomenon.** The layout is the same as Figure 3. Two ROIs (R1’ and R1”) are set to the anterior part of STG (aSTG) and the middle part of STG (mSTG) respectively. Each waveform in the upper-right panel shows the time course of the response amplitude in the ROIs. The radius of ROI was 10 mm. We calculated the waveform in a similar way to Fig. 3. Red bars under the waveforms indicate the volume-conducted responses suspected by shape and peak latency.


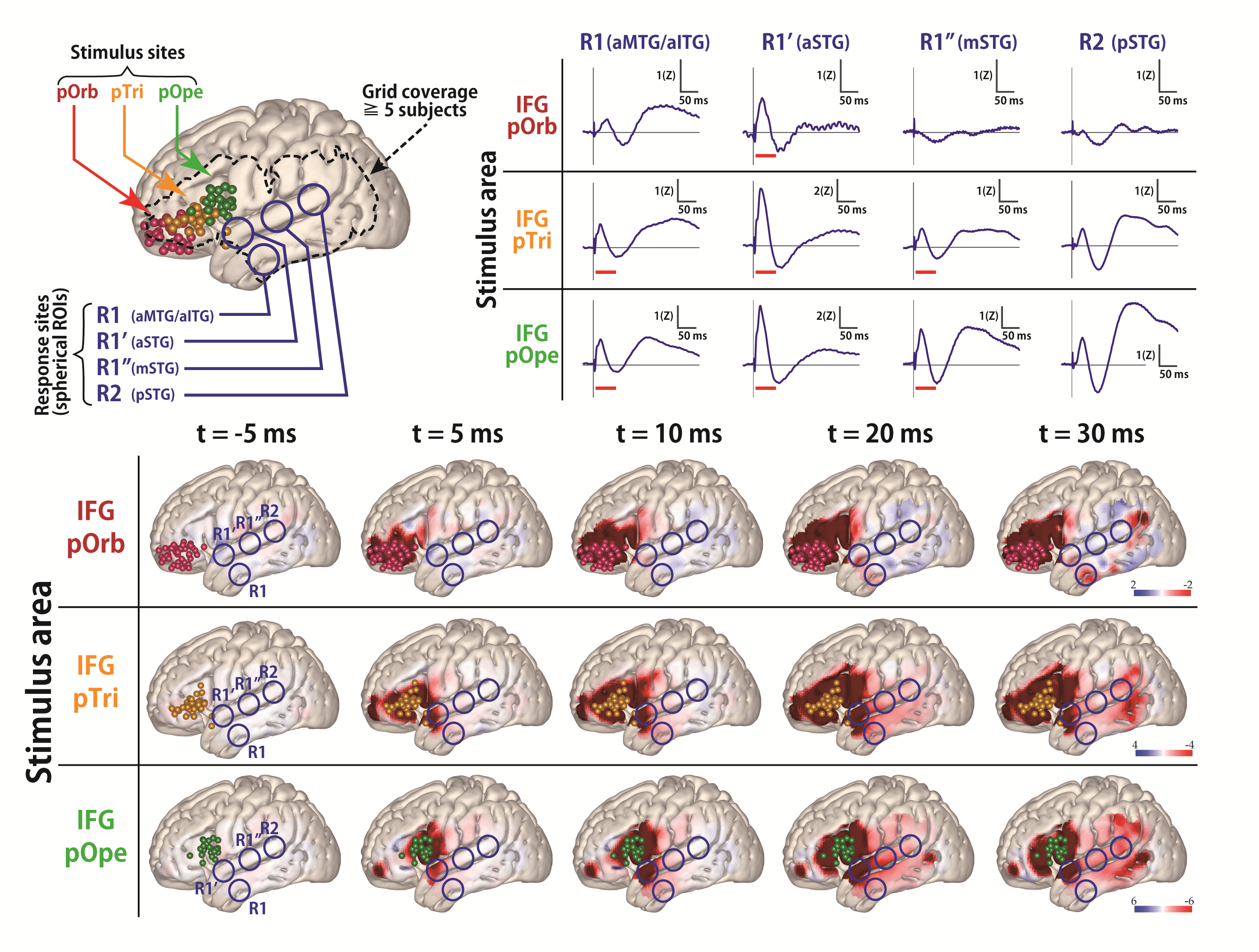


**Movie. “4D CCEP map”.**

Each movie represents the time-sequence of CCEP response by the stimulation of each part in IFG (pOrb, pTri or pOpe). To make a movie, we created images by 1 ms from 30 ms before the stimulus to 300 ms after the stimulus (total 331 images). Each image was derived from the standard brain surface painted according to the CCEP response distribution. The technical details in making the CCEP response distribution are described in the next section (titled as “**Technical details in making 4D CCEP map**”). To visualize the volume data on the brain surface model, we painted each vertex by the intensity of the nearest voxel according to the color scale, skipping those voxels which are covered by less than 5 subjects within 15 mm. Colored spheres indicates the location of the stimulus sites (red: IFG pOrb, orange: IFG pTri, green: IFG pOpe).

**Technical details in making “4D CCEP map”.**

We created the time-sequence of 3D volume image that represents the time course of the electric potential averaged across all subjects, which is called “averaged response map” hereafter. Averaging across subject is not simple because the location of electrodes are different by subjects. To solve that, we first accumulated the electric potential at all electrodes in the MNI space (voxel size: 2mm, isometric) through all subjects, plotting each recorded value (electric potential) at the voxel of electrode location. Then, we smoothed the volume (accumulated potential data) with Gaussian kernel (FWHM 6 mm, kernel size 10 mm) so as to embody the point data in 3D space. We set the FWHM as 6 mm taking the practical accuracy of the electrode determination into account. That is, we set FWHM as twice as much as the electrode diameter (3 mm), for we thought it is the lowest (humblest) precision when electrodes location are determined by the intraoperative picture. In this way, we obtained the volume image of the accumulation of recorded potentials which we call the “accumulated potential map”. Similarly, we obtained the “observation density map” by plotting “1” instead of the electric potential in each electrode. The “accumulated potential map” was divided by the “observation density map” to absorb the difference of the electrode density at the individual level. The result is called an averaged response map. Uneven electrode coverage was corrected by incorporating the electrode density information across the patients. We obtained an averaged response map for the stimulation of each subdivision of IFG (pOrb, pTri or pOpe). Note that we used all recorded data if the subject has several stimulus sites in the concerned subdivision of IFG (pOrb, pTri or pOpe). The calculation was skipped where the observation density was zero. We applied the spatial smoothing with Gaussian filter (FWHM 10 mm) to the “averaged response map”. We set the FWHM as 10 mm according to the inter-electrode distance, which is the expected resolution we expect when we perform functional mapping by grid electrodes.

**Technical details of reciprocality analysis**

A judgement of reciprocality was made from the CCEP database in the following three steps, which were implemented by the in-house MATLAB script. We checked the reciprocality of fronto-temporal (F🡪T) and fronto-parietal (F🡪P) connections separately. Taking the F🡪T connections as an example, our detailed method was as follows:

1. We extracted the temporal responses by frontal stimulation (= IFG pOrb stimulation in this study) from the CCEP database. The response electrode was defined in two ways; one is to select max responses (which showed the largest amplitude among the adjacent electrodes) and the other is to include all responses. We calculated the reciprocality rate for both types of responses. For each F🡪T connection, we repeated the below procedures (step 2 and 3) to judge the reciprocality.

2. For each F🡪T connection obtained in the previous step, we extracted the sessions of temporal lobe stimulation (searching for T🡪F connectivity) from the CCEP database. Note that we could not stimulate the temporal lobe through all of the F🡪T response electrodes due to the limited time in the operating room. Instead, we focused on the F🡪T response electrodes actually examined and calculated the rate of the reciprocality among them.

3. We made a judgement of reciprocality. When the T🡪F stimulation evoked a max response in at least one of the paired stimulus electrodes in the IFG pOrb, we considered there is a reciprocal connection between them.

Through the three steps, we counted the number of F🡪T responses (by IFG pOrb stimulation), the number of the stimulus sites via the response sites (searching for T🡪F connectivity) and the number of reciprocal connections. The reciprocality rate was calculated as the division of the number of reciprocal connections by the number of the stimulus sites searching for reciprocal (T🡪F) connectivity. We assessed reciprocality in six groups stratified by area (F🡪T vs. F🡪P) and the type of F🡪T (or F🡪P) response (max response, any response, or no response). We calculated the reciprocality rate not only for the response electrodes but for the no-response electrodes (i.e., those which did not show any response by IFG pOrb stimulation) to obtain the negative controls.
